# *Stellera chamaejasme* L. extract inhibits adipocyte differentiation through activation of the extracellular signal-regulated kinase pathway

**DOI:** 10.1371/journal.pone.0300520

**Published:** 2024-03-21

**Authors:** Jaegoo Yeon, Eunbin Kim, Badamtsetseg Bazarragchaa, Soo-Yong Kim, Jin Young Huh, Hyuntae Park, Sung-Suk Suh, Jong Bae Seo

**Affiliations:** 1 Department of Biosciences, Mokpo National University, Jeonnam, Republic of Korea; 2 Department of Biomedicine, Health & Life Convergence Sciences, BK21 Four, Biomedical and Healthcare Research Institute, Mokpo National University, Jeonnam, Republic of Korea; 3 Natural History Museum of Mongolia, Ulaanbaatar, Mongolia; 4 International Biological Material Research Center, Korea Research Institute of Bioscience and Biotechnology, Daejeon, Republic of Korea; 5 Department of Life Science, Sogang University, Seoul, Republic of Korea; 6 Department of Obstetrics & Gynecology, Korea University College of Medicine, Seoul, Republic of Korea; Northwest University, UNITED STATES

## Abstract

*Stellera chamaejasme* L. (SCL) is a perennial herb with demonstrated bioactivities against inflammation and metabolic dysfunction. Adipocyte differentiation is a critical regulator of metabolic homeostasis and a promising target for the treatment of metabolic diseases, so we examined the effects of SCL on adipogenesis. A methanol extract of SCL dose-dependently suppressed intracellular lipid accumulation in adipocyte precursors cultured under differentiation induction conditions and reduced expression of the adipogenic transcription factors PPARγ and C/EBPα as well as the downstream lipogenic genes fatty acid binding protein 4, adiponectin, fatty acid synthase, and stearoyl-CoA desaturase. The extract also promoted precursor cell proliferation and altered expression of the cell cycle regulators cyclin-dependent kinase 4, cyclin E, and cyclin D1. In addition, SCL extract stimulated extracellular signal-regulated kinase (ERK) phosphorylation, while pharmacological inhibition of ERK effectively blocked the inhibitory effects of SCL extract on preadipocyte differentiation. These results suggest that SCL extract contains bioactive compounds that can suppress adipogenesis through modulation of the ERK pathway.

## Introduction

*Stellera chamaejasme* L. (SCL) is a plant in the family *Thymelaeaceae* found in mountainous regions of Central Asia, China, Siberia, and South Asia [1]. While raw ingestion is known to cause diarrhea, vomiting, and even death, the roots (called Langdu) have long been used in China as a traditional treatment for dehydration, excess phlegm, and parasitic infection [1–4]. Recent preclinical investigations have also reported that various SCL extracts and constituents such as luteolin 7-O-glucoside can promote wound healing, quell inflammation, suppress the migration and induce the apoptotic death of cancer cells, and inhibit tumor angiogenesis [5–10]. In addition, there is evidence for possible efficacy against inflammatory and metabolic disorders [8, 11]. Adipocyte differentiation regulates metabolic homeostasis and dysregulation can contribute to obesity, a major risk factor for diabetes, heart disease, and stroke, so we examined the effects of SCL extract on the differentiation of adipocyte precursors and the underlying molecular signaling pathways.

Adipocytes regulate metabolic homeostasis not only by passive energy storage, but also through the regulated secretion of various hormones such as adiponectin and leptin [12–14]. Adipocyte differentiation is a complex process accompanied by coordinated changes in cell morphology, hormone sensitivity, and gene expression [13, 15–17]. The *in vitro* induction of adipocyte differentiation requires stimulation of intracellular cyclic-AMP accumulation (such as by the phosphodiesterase inhibitor 3-isobutyl-1-methylxanthine; IBMX) as well as activation of glucocorticoid and insulin receptors, which in combination upregulate expression of both the insulin receptor and insulin-like growth factor [13, 16, 18]. Treatment of confluent 3T3-L1 preadipocytes with this cocktail first results in the induction of one or two mitotic divisions, known as mitotic clonal expansion (MCE), followed by inhibition of proliferation and expression of various adipogenic transcription factors [13, 15–17]. Among the first of these to appear is CCAAT/enhancer-binding protein beta (C/EBPβ), which is required for the induction of MCE and expression of the downstream adipogenic transcription factors peroxisome proliferator-activated receptor gamma (PPARγ) and CCAAT/enhancer-binding protein alpha (C/EBPα) [19, 20]. Once expressed, PPARγ and C/EBPα upregulate the expression of hormones such as adiponectin and genes associated with the regulation of fatty acid storage, including fatty acid binding protein 4 (FABP4) [13, 21]. In addition, the transcription factor sterol regulatory element binding protein 1c (SREBP1c) activates the expression of enzymes and other proteins involved in the storage of triglycerides, such as fatty acid synthase (FAS), stearoyl-CoA desaturase, and acetyl-CoA carboxylase (ACC).

In the present study, we investigated if a methanol extract of SCL influences adipogenesis. We demonstrate that SCL extract can inhibit the differentiation of 3T3-L1 preadipocytes and primary stromal vascular cells (SVCs) by activating the ERK pathway, which in turn reduces the expression of PPARγ, C/EBPα, and downstream lipogenic target genes while promoting MCE.

## Materials and methods

### Preparation of SCL methanol extract

*Stellera chamaejasme* L. was collected in Handgaitiin Davaa district, Ulaanbaatar, Mongolia, and identified by Dr. Badamtsetseg Bazarragchaa at the National History Museum of Mongolia in June 2013. A voucher specimen of the retained material is preserved at the herbarium of the Korea Research Institute of Bioscience and BioTechnology (KRIBB, accession number KRIB 0049857). A methanol extract of the whole plant was procured from the International Biological Material Research Center at KRIBB, Daejeon, Republic of Korea (deposit number FBM188-029). Briefly, the leaves, shoots, roots, and flowers (30 g in total) were incubated in 1 L of 99.9% (v/v) methanol with repeating cycles of sonication (15 min) at 2 h intervals for 3 days at 45°C. The raw extract was filtered through non-fluorescent cotton and concentrated using a rotary evaporator (N-1000SWD, EYELA) at 45°C. Finally, 2.17 g of methanol extract was obtained by freeze-drying.

### Cell culture

The mouse embryonic fibroblast cell line 3T3-L1 was obtained from American Type Culture Collection (ATCC, Manassas, VA). The 3T3-L1 cell line was maintained in Dulbecco’s Modified Eagle’s Medium (DMEM; Welgene Inc., Deagu, Republic of Korea) supplemented with 10% bovine calf serum (Welgene), 100-U/mL penicillin, and 100-μg/mL streptomycin. Cells were maintained at 37°C in a humidified atmosphere with 10% CO_2_. To induce adipocyte differentiation, 3T3-L1 cells were seeded in 12-well culture plates at a density of 5.0 × 10^4^ cells/well, cultured for 2 days to reach confluence and until growth arrest occurred (defined as day 0, D0), and then cultured for 48 h (D0 to D2) in induction medium containing DMEM supplemented with 10% fetal bovine serum (FBS; Welgene), 0.5-mM IBMX (Sigma Aldrich, St Louis, MO), 1-μM dexamethasone (Sigma Aldrich), and 1-μg/mL insulin (Sigma Aldrich). Cells were then cultured in differentiation medium containing DMEM supplemented with 10% FBS and 1-μg/mL insulin. This medium was renewed every 48 h until day 6 (D6), as described previously [22].

### Animals

All animal experiments were performed in accordance with the National Institute of Health Guidelines for the Care and Use of Laboratory Animals as well as IACUC guidelines. All housing and experimental protocols were reviewed and approved by the Institutional Animal Care and Use Committee (IACUC) of Mokpo National University (Jeonnam, Republic of Korea; approval Nos. MNU-IACUC-2021-020 and -2023-006). Male, 12-week-old C57BL/6J mice were purchased from G-Bio (Gwangju, Republic of Korea) and housed under controlled conditions (23°C ± 2°C; relative humidity, 55% ± 10%; and 12-h/12-h light/dark cycle) with free access to standard rodent chow (PicoLab Rodent Diet 5053, Purina, St. Louis, MO) and ion-sterilized tap water. Mice were sacrificed by carbon dioxide inhalation.

### Isolation and differentiation of stromal vascular cells (SVCs)

Stromal vascular cells (SVCs, primary preadipocytes) containing preadipocytes were isolated from subcutaneous adipose tissue of wild-type C57BL/6J mice, as described previously [23]. Briefly, subcutaneous white adipose tissues were minced in a collagenase buffer (containing 2-mg/mL collagenase D) and digested by shaking (160 rpm) at 37°C for 40 min. After digestion of the adipose tissues, the solution was first filtered through a cell strainer (pore size, 100 μm) and centrifuged at 1,500 rpm for 5 min. The supernatant was then removed, and the pellet was resuspended and incubated using RBC lysis buffer (Thermo Fisher Scientific, Waltham, MA). Cells were filtered through a cell strainer (pore size, 40 μm) and briefly centrifuged to collect SVCs. The SVCs were cultured in complete medium (DMEM/F12 containing 10% FBS, penicillin/streptomycin (PS), and glutamine). For adipocyte differentiation, primary preadipocytes were first cultured to confluence in complete medium; then, differentiation was induced in DMEM supplemented with 10% FBS, PS, glutamine, and a differentiation cocktail comprising 0.5-mM IBMX, 1-μM dexamethasone, 1-μg/mL insulin, 0.2-mM indomethacin, and 1-μM rosiglitazone for 2 days. Subsequently, differentiated cells were cultured in DMEM containing 10% FBS, PS, glutamine, 1-μg/mL insulin, and 1-μM rosiglitazone. This medium was renewed every other day, as described previously [23].

### Cell viability assay

Cell viability was quantified using the WST-8 Cell Viability Assay Kit (BIOMAX, Seoul, Republic of Korea) according to the manufacturer’s protocol. Briefly, 3T3-L1 cells were seeded at 1 × 10^4^ cells per well in 96-well plates, cultured for 24 h or 6 days in DMEM containing 10% BCS and PS, treated with the indicated concentrations of SCL extracts for 24 h or every 2 days under standard culture conditions, and then incubated with WST-8 reagent at 37°C for 1 h. The absorbance of the samples at 450 nm was measured using an iMark^TM^ microplate reader (Bio-Rad Laboratories Inc., Hercules, CA); after background subtraction, an estimate of viable cell number was determined. Cell viability is expressed as a proportion (%) of viable cells in vehicle-treated control wells.

### Nile Red and Hoechst 33342 staining

Differentiated 3T3-L1 cells were carefully washed twice with Dulbecco’s phosphate buffered saline (DPBS), fixed in 3.7% formalin for 30 min, washed twice again in DPBS, and stained with 0.5-μg/mL Nile Red (Cayman Chemical, Ann Arbor, MI) and 1-μg/mL Hoechst 33342 (Invitrogen, Carlsbad, CA) for 10 min, as described earlier [22]. The lipid droplet staining and cell morphology were examined using a fluorescence microscope (NIB410, Nexcope, Ningobo, China) at ×200 magnification. All images were analyzed using ImageJ (NIH, Bethesda, MD).

### Quantitative real-time PCR

Total RNA from the treated cells was isolated using RiboEx^TM^ reagent (GeneAll Biotechnology, Seoul, Republic of Korea) and quantified using a NanoPhotometer N60 (Implen, CA, USA). Subsequently, 1 μg of RNA was reverse transcribed into cDNA using the ReverTra Ace^TM^ qPCR RT kit (Toyobo, Osaka, Japan) and a T100 Thermal Cycler (Bio-Rad). Quantitative real-time PCR (qRT-PCR) was conducted using a CFX Connect Real-Time PCR system (Bio-Rad) and real-time PCR Master Mix, including SFCgreen I (BIOFACT, Dajeon, Republic of Korea). After 40 cycles, the specificity of the PCR reaction was confirmed through melting curve analysis and gene expression was quantified by comparing CT values with those of standard 36B4 gene. Most of primer sequences used in the current experiments had been used in our previous study [24]. The sequences of the remaining primers are as follows: CDK2, (F) 5’-CCC TTC CCA AAG CCC TTT TC-3’ and (R) 5’-GAA GAG GGG AAG CTG GT-3’; CDK4, (F) 5’-ATG GCT GCC ACT CGA TAT GAA-3’ and (R) 5’- TCC ATT AGG AAC TCT CAC AC-3’; Cyclin D1, (F) 5’-GCG TAC CCT GAC ACC AAT CTC-3’and (R) 5’-CTC CTC TTC GCA CTT CTG CTC-3’; Cyclin E,: (F) 5’-GTG GCT CCG ACC TTT CAG TC-3’ and (R) 5’-CAC AGT CTT GTC AAT CTT GGC A-3’; E2F3, (F) 5’-CGG TCT GCT CAC CAA GAA GT-3’ and (R) 5’-CCT CTT CTG CAC CTT GAG CA-3’; C/EBPβ, (F) 5’-CAA CCT GGA GAC GCA GCA CAA G-3’ and (R) 5’-GCT TGA ACA AGT TCG GCA GGG T-3’.

### Western blotting analysis

After the indicated treatment, cells were lysed in Mammalian Protein Extraction Reagent (Thermo Scientific, Rockford, IL) containing Xpert protease inhibitor cocktail (GenDEPOT, Baker, TX) and Xpert phosphatase inhibitor cocktail (GenDEPOT) and centrifuged at 12,000 rpm for 10 min at 4°C. Protein samples were separated using SDS-PAGE and subsequently transferred onto PVDF membranes. The membranes were then blocked by immersion in blocking buffer consisting of 5% bovine serum albumin in TBST (TBST is 20-mM Tris-HCl, 150-mM NaCl, 0.2% Tween 20, pH 7.4) at RT for 1 h on a shaker. The membranes were washed three times with TBST and then incubated with an anti-PPARγ antibody (Cell Signaling Technology, Danvers, MA, Cat. No. 2443), anti-adiponectin antibody (Thermo Scientific, Cat. No. MA1-054), anti-ERK antibody (Cell Signaling Technology, Cat. No. 91025S), anti-phosphoERK antibody (Cell Signaling Technology, Cat. No. 4370S), and an anti-HSP 90 antibody (Santa Cruz Biotechnology, Dallas, TX, Cat. No. SC-13119) at 4°C overnight. After washing with fresh TBST, membranes were incubated with rabbit or mouse IgG conjugated to horseradish peroxidase (Bio-Rad; 1:5,000 dilution). Protein bands were visualized using ECL reagent (Bio-Rad) and an iBright CL1500 imaging system (Thermo Scientific).

### Cell cycle analysis

Cell cycle phase distribution was measured by propidium iodide (PI) staining and flow cytometry. Cells were detached from the plate using Trypsin-EDTA Solution (Welgene), washed with DPBS, fixed in 70% ethanol at −20°C for 16 h, washed twice in DPBS, treated with RNase A (100 μg/mL) (BIOFACT, Cat. No. PE290-25h), and stained with 2 μg/mL PI (Thermo Scientific, Cat. No. P3566). Staining profiles indicative of cell cycle stage distribution were measured using a CytoFLEX flow cytometer (Beckman Coulter, Brea, CA, USA) and CytExpert software (Beckman Coulter). Results are presented as content histograms, with number of cells plotted on the y-axis and DNA content as measured by PI fluorescence on the x-axis.

### Statistical analysis

All results are presented as mean ± standard error of the mean (SEM), while *n* denotes the number of culture wells analyzed. Two treatment groups were compared by unpaired two-tailed Student’s *t*-test using Microsoft Excel, while more than two groups were compared by analysis of variance with post hoc multiple comparisons tests using GraphPad Prism. A *p* < 0.05 was considered statistically significant for all tests.

## Results

### *Stellera chamaejasme* L. extract inhibits adipocyte differentiation

**Fig 1:**
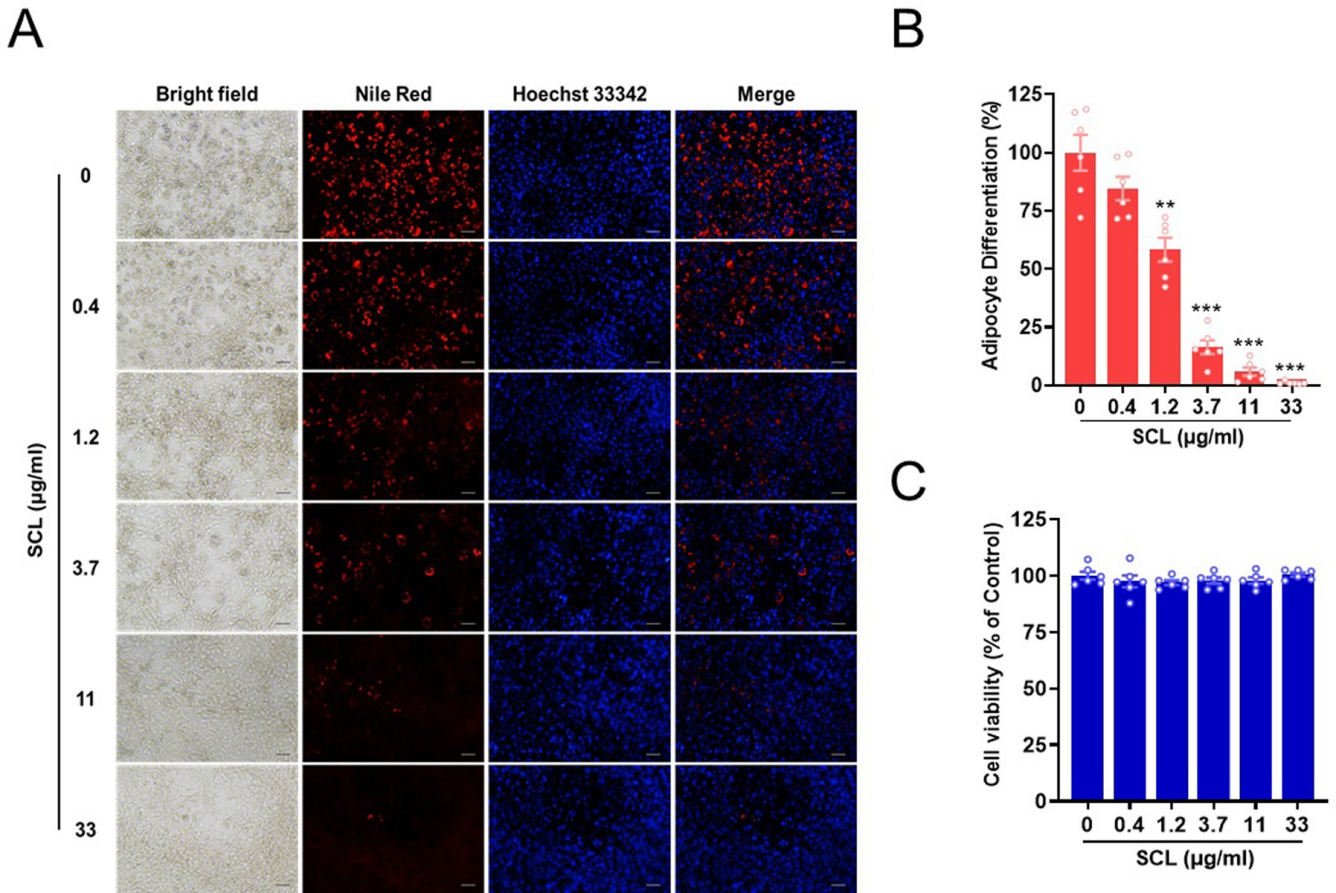
*Stellera chamaejasme* L. methanol extract suppressed the induced differentiation of 3T3-L1 preadipocytes without substantial cytotoxicity. (**A**) 3T3-L1 preadipocytes were cultured in differentiation medium for 6 days in the presence of *Stellera chamaejasme* L. (SCL) methanol extract or vehicle (control). Cells were then stained for lipid accumulation using Nile Red, while nuclei were counterstained with Hoechst 33342. Images were acquired by epifluorescence microscopy. The scale bar represents 100 μm. (**B and C**) Nile Red staining intensity and cell viability were quantified using ImageJ software (*n* = 6 wells per treatment). All results are presented as the mean ± SEM of two independent experiments. **, *p* < 0.01; ***, *p* < 0.001 *versus* the control group.

The cytotoxicity of SCL extract on both 3T3-L1 preadipocytes and primary preadipocytes was first examined using the WST-8 viable cell counting assay (S1 Fig). Treatment with SCL extract for 24 h or 6 days did not significantly impact cell viability compared to vehicle (DMSO) treated controls (S1 Fig). However, SCL extract exhibited a dose-dependent decrease in intracellular lipid accumulation, a phenotypic hallmark of adipocyte differentiation [16, 25], as measured by Nile Red fluorescence staining in 3T3-L1 preadipocytes cultured under differentiation induction conditions for 6 days without cytotoxicity (Fig 1).

**Fig 2:**
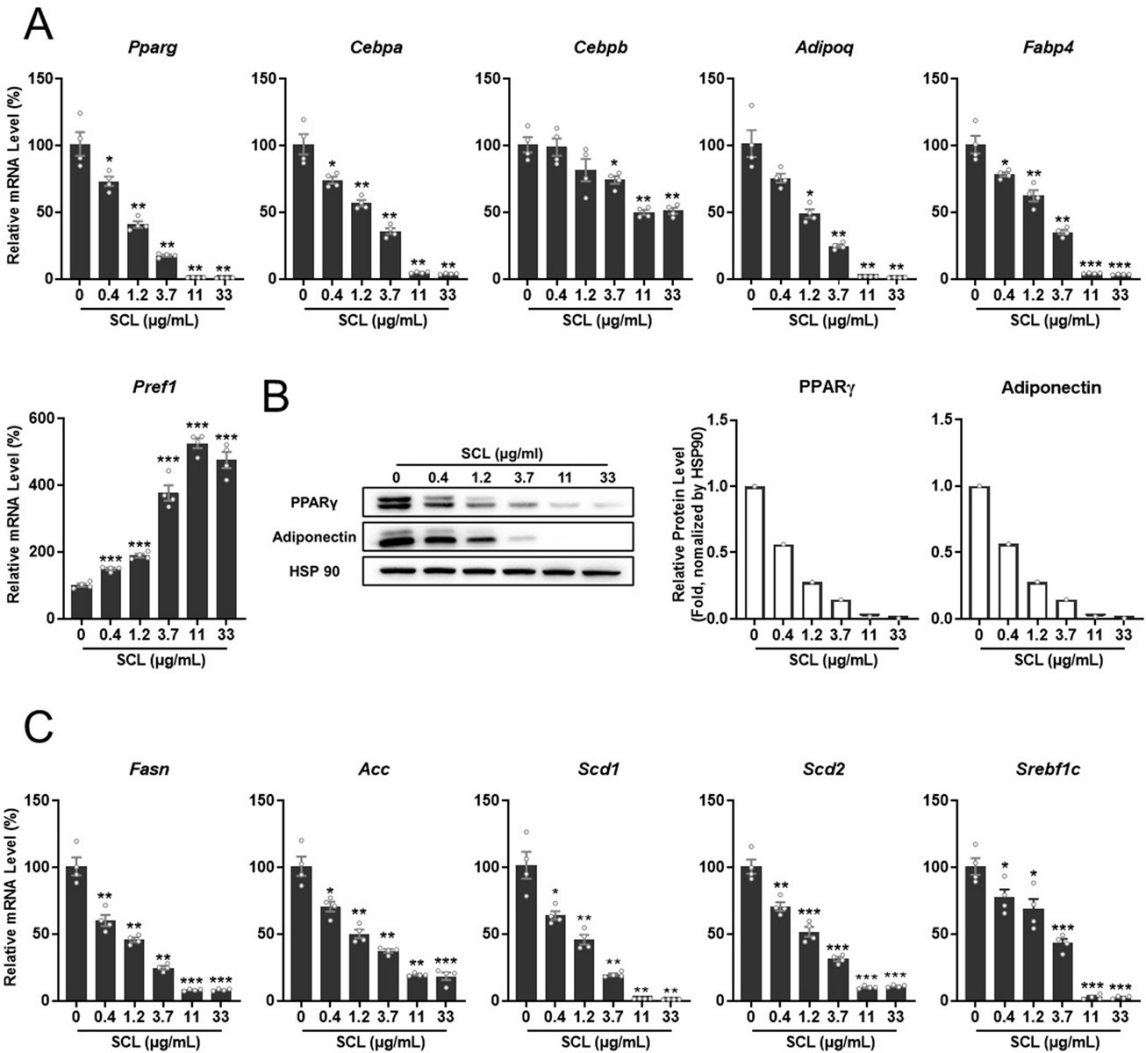
*Stellera chamaejasme* L. methanol extract suppressed the expression of adipogenic and lipogenic genes in 3T3-L1 cells cultured under differentiation induction conditions. **−**Cells were cultured for 6 days in differentiation medium plus SCL or vehicle. (**A**) The mRNA expression levels of adipogenic genes were analyzed by quantitative real-time PCR (qRT-PCR). (**B**) Expression levels of PPARγ and Adiponectin proteins were determined by Western blotting. (**C**) The mRNA expression levels of lipogenic genes were analyzed by qRT-PCR. All results are presented as the mean ± SEM of two independent experiments (*n* = 4 wells per treatment). *, *p* < 0.05; **, *p* < 0.01; ***, *p* < 0.001 *versus* the control group.

To examine the molecular mechanisms underlying the suppression of lipid accumulation by SCL extract, changes in the expression levels of adipogenic and lipogenic genes were measured at the mRNA and protein levels by quantitative real-time (qRT)-PCR and Western blotting, respectively. The mRNA expression levels of multiple adipogenic genes (*Pparg*, *Cebpa*, *Cebpb*, *Adipoq*, and *Fabp4*) were significantly and dose-dependently reduced by SCL extract, while mRNA expression of the preadipocyte marker *Pref1* was enhanced compared to vehicle-treated controls (Fig 2A). Consistent with qRT-PCR results, the protein expression levels of PPARγ and Adiponectin were dose-dependently downregulated by SCL extract (Fig 2B). Quantitative RT-PCR also revealed reduced mRNA expression of the lipogenic genes *Fasn*, *Acc*, *Scd1*, *Scd2*, and *Srebf1c* (Fig 2C), indicating that this extract could inhibit adipocyte differentiation and mature cell function.

### SCL extract acts at multiple stages of adipocyte differentiation

**Fig 3:**
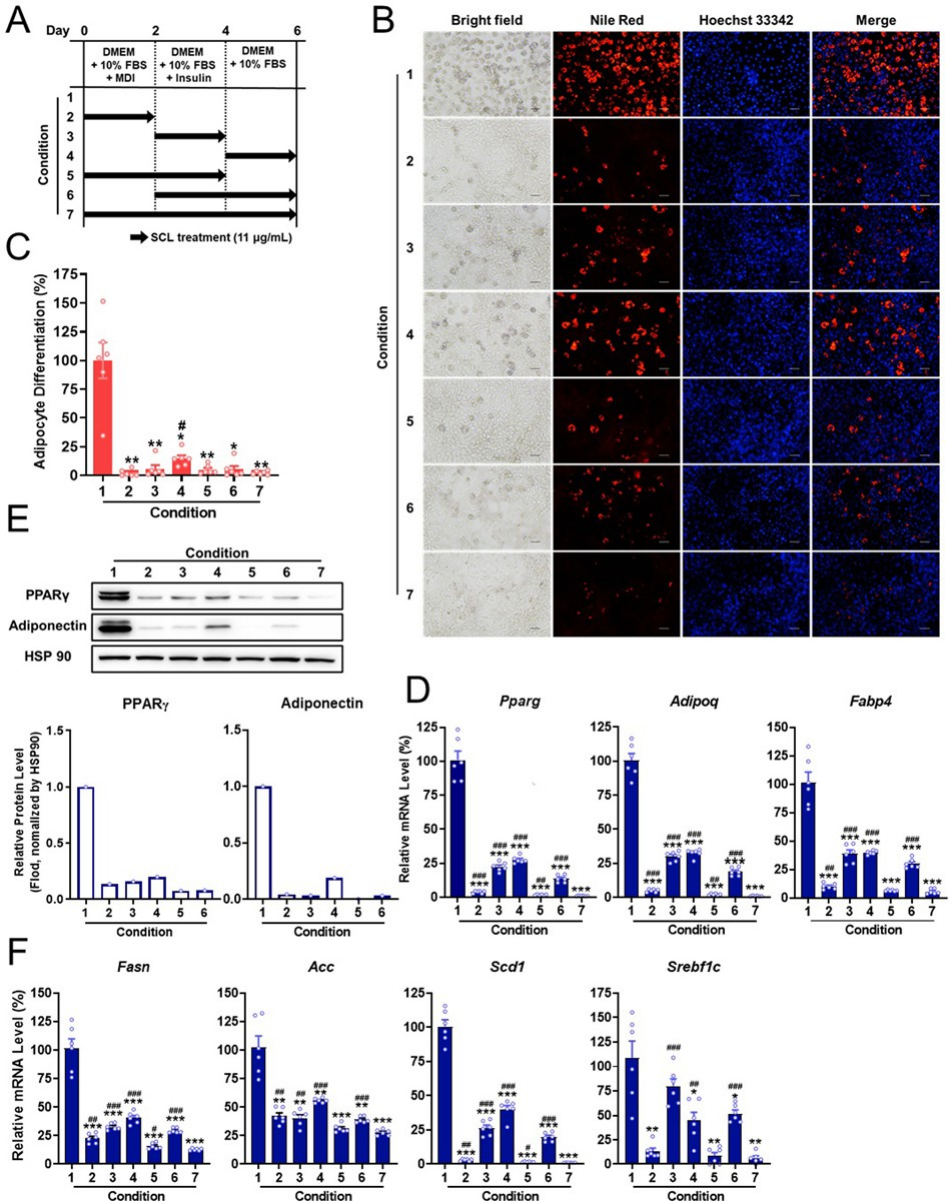
*Stellera chamaejasme* L. methanol extract interferes with multiple stages of adipocyte differentiation. (**A**) Schematic representation of the experiment. Cultures of 3T3-L1 cells were treated with SCL at different times during the 6-day differentiation induction period to block specific stages. The fresh medium contained SCL extract that was changed every other day during adipogenesis. (**B**) After the differentiation induction period, cells were stained with Nile Red to assess adipogenesis and nuclei were counterstained with Hoechst 33342. Images were acquired by epifluorescence microscopy. The scale bar represents 100 μm. (**C**) The reduction in lipid accumulation differed according to the SCL extract exposure period (Condition) (*n* = 6 wells per treatment). (**D** and **F**) Extract treatment during the induction protocol also reduced the mRNA expression levels of adipogenic (**D**) and lipogenic (**F**) genes as estimated by qRT-PCR. (**E**) Expression levels of PPARγ and Adiponectin proteins were determined by Western blotting. All results are presented as the mean ± SEM of two independent experiments (*n* = 4 wells per treatment). *, *p* < 0.05; **, *p* < 0.01; ***, *p* < 0.001 *versus* the control group (condition 1). ^#^, *p* < 0.05; ^##^, *p* < 0.01; ^###^, *p* < 0.001 *versus* the SCL treatment group for 6 day (condition 7).

To identify the stage of adipocyte differentiation disrupted by SCL extract, post-confluent 3T3-L1 preadipocytes were treated with 11 μg/mL SCL extract at different timepoints during induction as illustrated in Fig 3A. All conditions of SCL treatment significantly inhibited adipocyte differentiation, as shown in Fig 3B and 3C. Overall treatment on days 0–2 (Condition 2) and days 0–4 (Condition 5) of induction culture inhibited differentiation about as effectively as treatment for all 6 days of induction (Condition 7). These three treatment conditions also significantly downregulated the mRNA expression levels of adipogenic and lipogenic genes and the protein expression levels of PPARγ and Adiponectin (Fig 3D–3F). Moreover, extract treatment on days 2–4 (Condition 3), days 4–6 (Condition 4), and days 2–6 (Condition 6) still suppressed differentiation, albeit less strongly than Conditions 2, 5, and 7. These results suggest that SCL extract exerts an anti-adipogenic effect during multiple stages of differentiation.

### SCL extract inhibits the adipogenic differentiation of primary stromal vascular cells (SVC)

**Fig 4:**
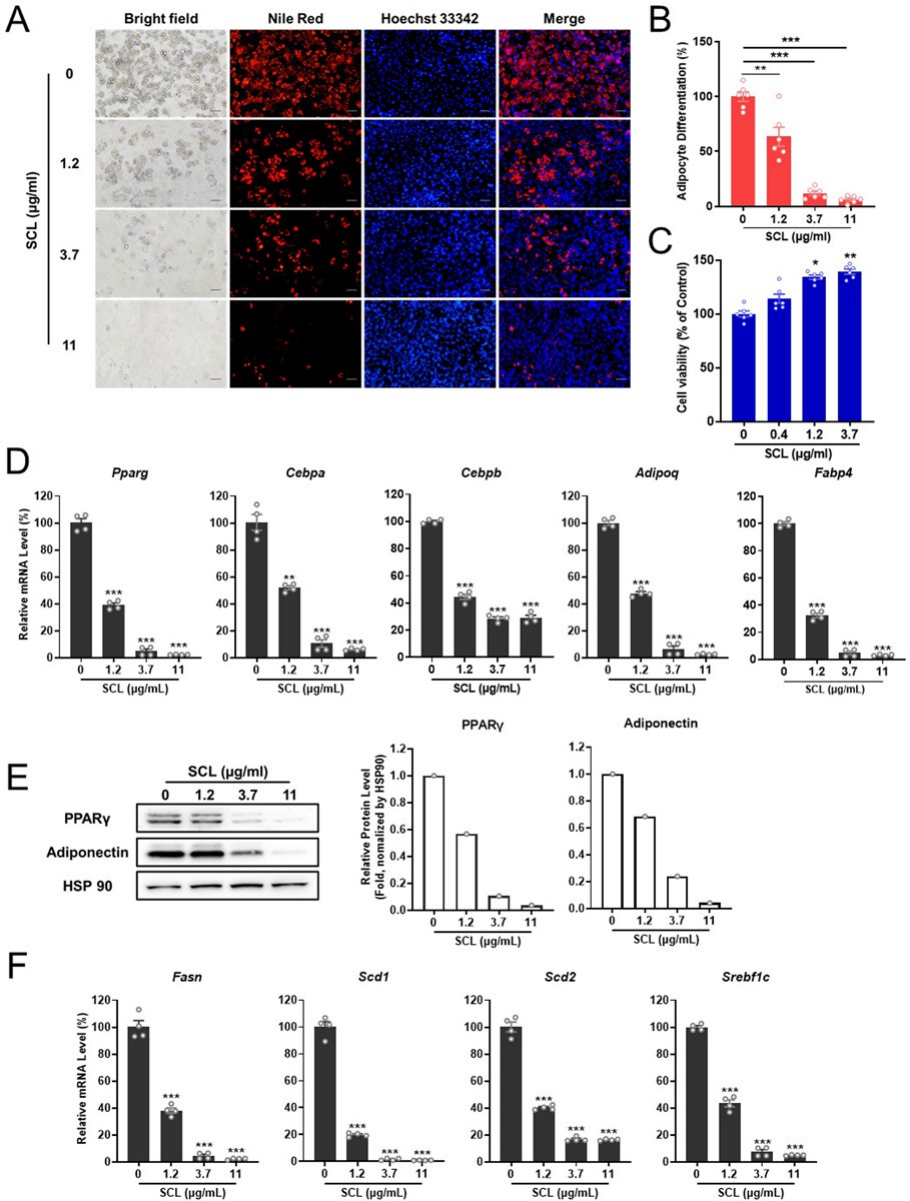
*Stellera chamaejasme* L. methanol extract inhibited the adipogenic differentiation of primary SVCs. Cells were cultured under differentiation induction conditions for 6 days in the absence (control) or presence of various SCL extract concentrations. (**A**) After the differentiation protocol, cells were stained with Nile Red to assess lipid accumulation and nuclei were counterstained with Hoechst 33342. Images were acquired under epifluorescence microscopy. The scale bar represents 100 μm. (**B**) The SCL extract reduced lipid accumulation in SVC-derived adipocytes as measured by Nile Red staining (*n* = 6 wells per treatment). (**C**) The number of cells was counted by Hoechst 333342 staining (*n* = 6 wells per treatment). (**D** and **F**) The SCL extract also reduced the mRNA expression levels of adipogenic and lipogenic genes as estimated by qRT-PCR. (**E**) PPARγ and Adiponectin protein expression levels were reduced by SCL extract as evidenced by Western blotting. All results are presented as the mean ± SEM of two independent experiments (*n* = 4 wells per treatment). **, *p* < 0.01; ***, *p* < 0.001 *versus* the control group.

To investigate if these anti-adipogenic effects extend to primary differentiating adipocytes, we repeated these experiments on primary SVCs from mouse inguinal white adipose tissue (Fig 4). Again, treatment with SCL extract significantly and dose-dependently reduced lipid accumulation without cell toxicity as evidenced by Nile Red and Hoechst 333342 staining, respectively, (Fig 4A–4C), the mRNA expression levels of core adipocyte-specific marker genes *(Pparg*, *Cebpa*, *Cebpb*, *Adipoq*, and *Fabp4*) (Fig 4D), the protein levels of PPARγ and Adiponectin (Fig 4E), and the mRNA levels of the lipogenic genes *Fasn*, *Acc*, *Scd1*, *Scd2*, and *Srebf1c* (Fig 4F).

### SCL extract inhibits adipocyte differentiation via modulation of both adipocyte-specific transcription factor expression and mitotic clonal expansion (MCE)

**Fig 5:**
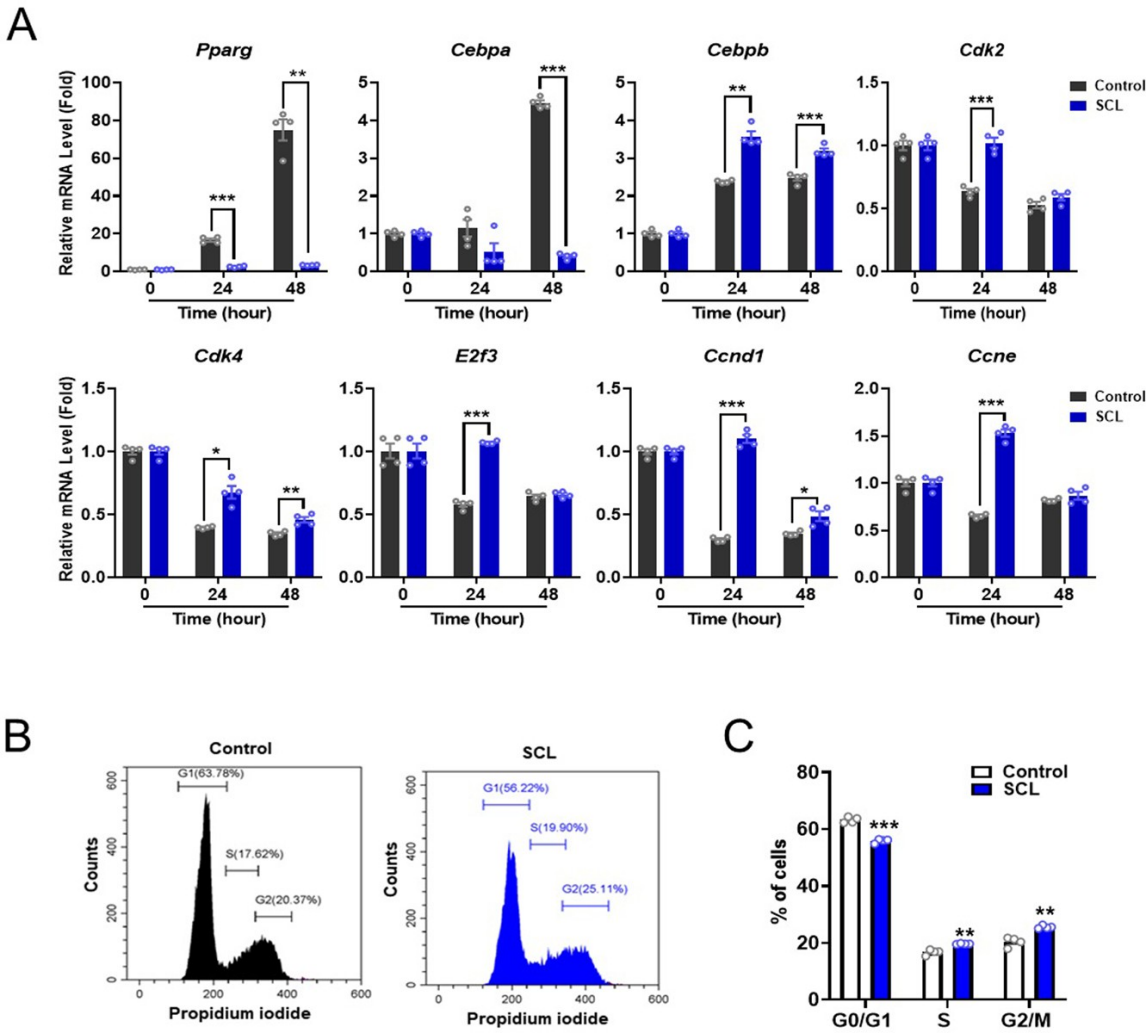
The SCL extract enhanced mitotic clonal expansion of 3T3-L1 cells during the early period of adipocyte differentiation. (**A**) 3T3-L1 preadipocytes were cultured under differentiation induction conditions for 0, 24, and 48 h in the absence (control) or presence of SCL extract. The extract reduced the mRNA expression levels of adipocyte-specific transcription factors and enhanced expression of cell cycle-related genes as estimated by qRT-PCR (*n* = 4 wells per treatment). (**B**) Representative flow cytometry images showing the cell cycle phase distribution of 3T3-L1 cells cultured under differentiation induction conditions in the absence and presence of SCL extract. (**C**) Quantitative analysis of flow cytometry results. The extract enhanced the proportions of cells in S phase and G2/M phase. All results are presented as the mean ± SEM from two independent experiments. *, *p* < 0.05; **, *p* < 0.01; ***, *p* < 0.001 *versus* each control group.

To investigate the cellular and molecular mechanisms underlying the suppression of adipocyte differentiation by SCL extract, we examined adipocyte-specific transcription factor expression and MCE during the early stages of induction in 3T3-L1 preadipocytes. As shown in Fig 5A, SCL extract inhibited the mRNA expression of *Pparg* and *Cebpa* genes, but increased mRNA levels of the *Cebpb* gene. Although C/EBPβ has an important role in MCE during adipogenesis [19], the excessive increase in *Cebpb* expression might be owing to the stimulation of cell proliferation by the SCL extract. In addition, SCL extract enhanced the mRNA expression levels of cell cycle-related genes encoding *Ccne*, *Ccnd1*, *E2f3*, cyclin-dependent kinase 2 (*Cdk2*), and *Cdk4* (Fig 5A). Interestingly, the decreased expression of *E2f3* and *Ccne* at 48 h appears to be owing to the inhibition of cell–cell contacts. The SCL extract also altered the cell cycle phase distribution as evidenced by PI staining and flow cytometry (Fig 5B and 5C). Compared to vehicle-treated controls, SCL extract-treated cultures exhibited a greater proportion of cells in S phase (16.7% ± 0.49% vs. 19.7% ± 0.07%) and G2/M phase (20.4% ± 0.85% vs. 25.6% ± 0.27%) and a lower proportion in G0/G1 phase (63.2% ± 0.52% vs. 55.9% ± 0.35%), suggesting an enhanced rate of cell cycle progression. Consistent with Fig 5A, SCL extract increased mRNA expression of the cell cycle-related proteins *CCNE*, *CCND1*, *E2f3*, and *Cdk4* (S2 Fig) and dose-dependently enhanced the proliferation rate of 3T3-L1 preadipocytes as measured by WST-8 viable cell counting assay (S2 Fig). Taken together, these results suggest that both the downregulation of adipocyte-specific transcription factors and enhanced MCE contribute to the suppression of adipocyte differentiation by SCL extract.

### SCL extract inhibits adipocyte differentiation via activation of the extracellular signal-regulated kinase (ERK) signaling pathway

**Fig 6:**
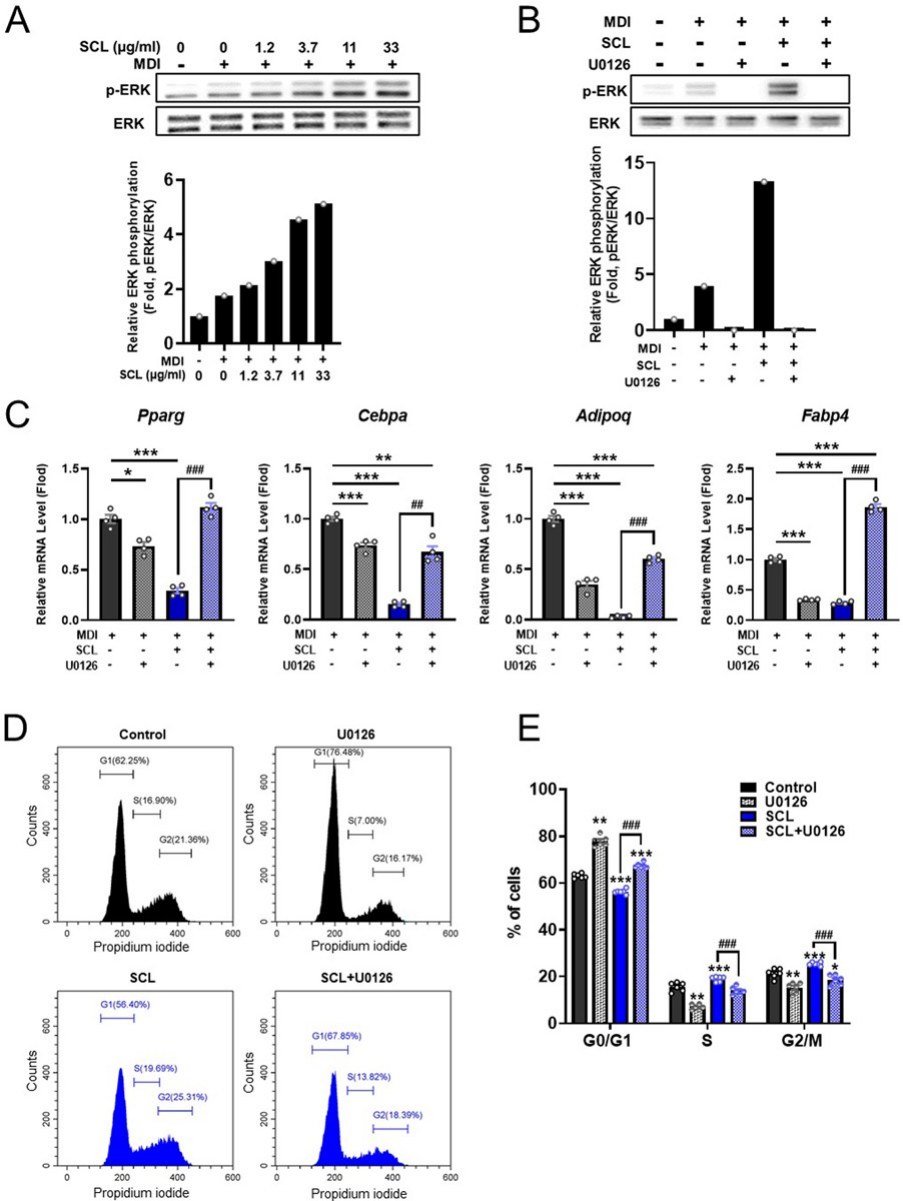
The SCL extract blocked adipocyte differentiation and promoted mitotic clonal expansion of preadipocytes through activation of the ERK pathway. (**A**) 3T3-L1 preadipocytes were cultured in induction medium containing various concentrations of SCL for 15 min. (**B**) 3T3-L1 preadipocytes were cultured in induction medium containing 33-μg/mL SCL for 15 min in the absence or presence of the ERK inhibitor U0126 (10 μM). Total ERK and phosphorylated (p)-ERK protein expression levels as determined by Western blotting. (**C**) The ERK inhibitor U0126 (10 μM) reversed the inhibitory effect of SCL (33 μg/mL) on adipocyte marker gene expression as determined by qRT-PCR. (**D** and **E**) The ERK inhibitor U0126 (10 μM) reversed the effect of SCL extract (33 μg/mL) on mitotic clonal expansion. (**D**) Representative flow cytometry images showing the cell cycle phase distribution. (**E**) Quantitative analysis of the cell cycle phase distribution. Cotreatment with U0126 reversed the SCL extract-induced increases in S and G2/M phase cells. All results are presented as the mean ± SEM of two independent experiments. *, *p*<0.05; **, *p* < 0.01, ***, *p* < 0.001 *versus* each control group ^#^, *p* < 0.01, ^###^, *p* < 0.001.

It has been reported that ERK signaling pathways are involved in adipocyte differentiation at the early stage [26] and that SCL extract promotes ERK phosphorylation (activation) in HaCaT keratinocytes [8]. Thus, we evaluated whether SCL extract can regulate the ERK signaling pathway in preadipocytes and early-stage differentiating adipocytes by monitoring ERK phosphorylation. Consistent with a previous report [8], SCL extract significantly and dose-dependently stimulated ERK phosphorylation in both 3T3-L1 preadipocytes (S3 Fig) and 3T3-L1 cells under adipocyte differentiation culture conditions (Fig 6A and 6B). The SCL-activated ERK was attenuated by the ERK-specific inhibitor U0126 (Figs 6B and S3). Moreover, the treatment of U0126 (Figs 6A and 6B and S3) reversed the inhibitory effects of SCL extract on early-stage adipocyte differentiation (Fig 6C). By day 2 of induction in the IBMX-dexamethasone-insulin (MDI) cocktail, SCL extract reduced mRNA expression levels of the differentiation-associated transcription factors *Pparg* and *Cebpa* by 29% ± 2.8% and 15% ± 1.5%, respectively, of the expression levels measured in MDI medium without SCL extract. Further, mRNA expression levels of the adipocyte marker genes *Adipoq* and *Fabp4* were downregulated by 3% ± 0.6% and 29% ± 1.5%, respectively, compared to the control group. Again, these inhibitory effects were completely or partially reversed by U0126 treatment (*Pparg*: 112% ± 4.3% of expression in MDI medium with SCL extract alone; *Cebpa*: 67% ± 5.7%; *Adipoq*: 60% ± 1.8%; *Fabp4*: 187% ± 4.4%). Flow cytometry and PI staining also revealed that cotreatment with U0126 reversed the increase in S phase cells and G2/M phase cells induced by SCL (S phase, control: 15.6% ± 0.78%, SCL: 19.0% ± 0.44%, SCL+U0126: 13.9% ± 0.69%; G2/M phase: control: 21.4% ± 0.83%, SCL: 25.5% ± 0.31%, SCL+U0126: 18.6% ± 0.90%) in Fig 6D and 6E. In addition, consistent with a previous report [26], the U0126 treatment alone reduced the expression of these adipogenic transcription factors and marker genes (Fig 6C) and the populations of S and G2/M phase cells (Fig 6D and 6E) compared with the control group. These results indicate that SCL extract suppresses adipocyte differentiation and promotes MCE through ERK activation.

## Discussion

We demonstrate that SCL extract can inhibit adipocyte differentiation by downregulating expression of core adipogenic transcription factors (PPARγ and C/EBPα) and target lipogenic genes through activation of the ERK signaling pathway. We suggest that this SCL methanol extract may contain bioactive factors that exert influential effects on metabolic homeostasis.

*Stellera chamaejasme* L. (SCL) has been used in traditional Chinese medicine to treat tuberculosis, tumors, and psoriasis among other disorders, and more recent studies have demonstrated potent anti-inflammatory, analgesic, antineoplastic, and wound healing properties [5–11, 27]. For instance, Kim and colleagues reported that SCL extract and its primary bioactive constituents facilitated the healing of skin lesions in rodents by boosting keratinocyte migration and collagen expression, while concurrently mitigating inflammatory cytokine expression and prostaglandin E2 (PGE2) production [8]. Moreover, they reported that ERK activation contributed to the enhanced migration of keratinocytes [8], in accord with the current finding that SCL extract dose-dependently enhanced ERK phosphorylation (activation) in both 3T3-L1 preadipocytes and SVCs in the early stages of adipocyte differentiation. The profound impact of SCL on lipid accumulation and on adipogenic and lipogenic gene expression was also counteracted by pharmacological ERK inhibition. Further studies are warranted to identify the bioactive constituents mediating these anti-adipogenic effects and the contributions of specific ERK pathways.

Although numerous studies have delineated the role of ERK in adipocyte differentiation, the reported outcomes have varied [26, 28–35]. Some indicate that ERK activation hampers adipocyte differentiation [33–35], whereas others find it synchronous with mitotic signaling or adipogenic differentiation [26, 30]. One potential explanation for these contradictory findings is that ERK activity has unique effects during different stages of adipogenesis. In the early stages, ERK appears to activate proliferation [19, 29], which constrains further differentiation, while in the late stage, ERK phosphorylates PPARγ and subsequently reduces adipogenic gene activation [28, 29, 31, 32]. Therefore, it is possible that ERK activity may promote or suppress differentiation under certain conditions. However, the results presented here support only a suppressive effect of ERK on adipocyte differentiation and a facilitatory effect on clonal expansion of preadipocytes (Figs 5 and 6). The mRNA expression of PPARγ has been shown to be negatively regulated by ERK activation. The mRNA expression levels of PPARγ and C/EBPα as well as the expression levels of many downstream target genes were significantly reduced in SCL extract-treated cells during culture under adipocyte differentiation conditions, and this anti-adipogenic effect was reversed by the ERK inhibitor U0126. Interestingly, when ERK was inhibited by treatment with U0126 alone, adipocyte differentiation was attenuated, similar to a previous studies [26]. This result suggests that a certain level of ERK activation is required at the initiation of adipocyte differentiation. Taken together, these findings strongly suggest that SCL extract inhibits adipocyte differentiation and promotes preadipocyte proliferation by excessively activating ERK, which in turn blocks the transactivational activities of core adipogenic transcription factors. Elucidating the precise molecular mechanism by which SCL extract components activate ERK and subsequently reduce PPARγ may provide clues for the therapeutic manipulation of adipogenesis in metabolic diseases.

## Supporting information

S1 Fig No cytotoxic effects on 3T3-L1 preadipocytes and primary preadipocytes by SCL extract. (**A**) 3T3-L1 preadipocytes were treated with SCL extract for 24 h and cell viability was measured using WST-8 colorimetric assay (*n* = 9 wells). (**B**) 3T3-L1 cells and (**C**) primary preadipocytes (SVC) were incubated with SCL extract for 6 days (*n* = 6 wells). Fresh medium was exchanged with SCL extract every other day. The results are presented as mean ± SEM of two independent experiments.
(TIF)

S2 Fig Enhancement of the proliferation of 3T3-L1 preadipocytes by SCL extract. 3T3-L1 preadipocytes were cultured for 24, 48, or 72 h in the absence (control) or presence of 33-μg/mL SCL. (**A**) The effect of SCL on the expression of cell cycle-related genes (*n* = 6). (**B**) Proliferation rate was analyzed using a WST-8-based colorimetric assay kit (*n* = 18). All results are presented as mean ± SEM from two independent experiments. *, *p* < 0.05; **, *p* < 0.01; ***, *p* < 0.001 vs. each control group.
(TIF)

S3 Fig Activation of ERK pathway in naïve 3T3-L1 preadipocytes by SCL extract. (**A**) 3T3-L1 preadipocytes were treated with various concentrations of SCL for 15 min. (**B**) 3T3-L1 preadipocytes were treated with 33-μg/mL SCL for 15 min in the absence or presence of ERK inhibitor U0126 (10 μM). Total ERK and phosphorylated (p)-ERK protein expression levels, as determined by western blotting.
(TIF)

S4 Fig Original western blot gel image data. (PDF)
