# Supplementary figures and images for: Stellera chamaejasme L. extract inhibits adipocyte differentiation through activation of the extracellular signal-regulated kinase pathway
Source: PLoS One. 2024 Mar 21;19(3):e0300520. doi: 10.1371/journal.pone.0300520 (PMC10956757; doi:10.1371/journal.pone.0300520)

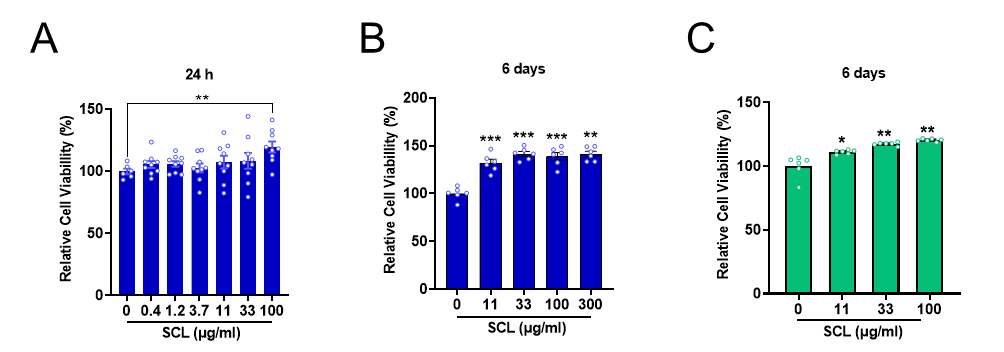

Supplement: S1 Fig — (A) 3T3-L1 preadipocytes were treated with SCL extract for 24 h and cell viability was measured using WST-8 colorimetric assay (n = 9 wells). (B) 3T3-L1 cells and (C) primary preadipocytes (SVC) were incubated with SCL extract for 6 days (n = 6 wells). Fresh medium was exchanged with SCL extract every other day. The results are presented as mean ± SEM of two independent experiments. (TIF) [file pone.0300520.s001.tif]

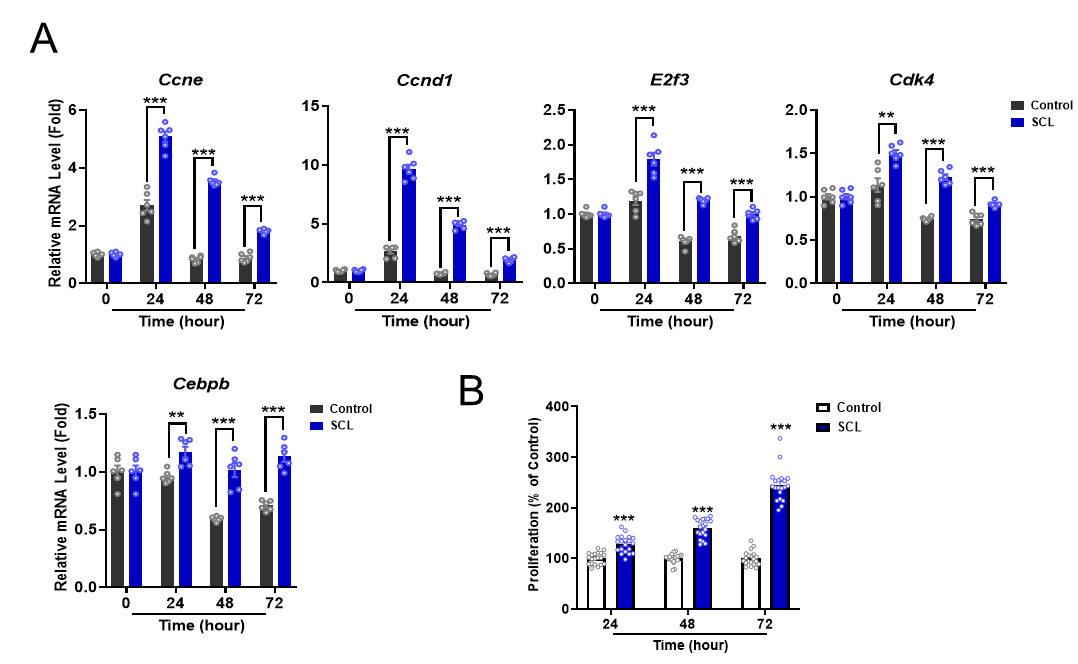

Supplement: S2 Fig — 3T3-L1 preadipocytes were cultured for 24, 48, or 72 h in the absence (control) or presence of 33-μg/mL SCL. (A) The effect of SCL on the expression of cell cycle-related genes (n = 6). (B) Proliferation rate was analyzed using a WST-8-based colorimetric assay kit (n = 18). All results are presented as mean ± SEM from two independent experiments. *, p < 0.05; **, p < 0.01; ***, p < 0.001 vs. each control group. (TIF) [file pone.0300520.s002.tif]

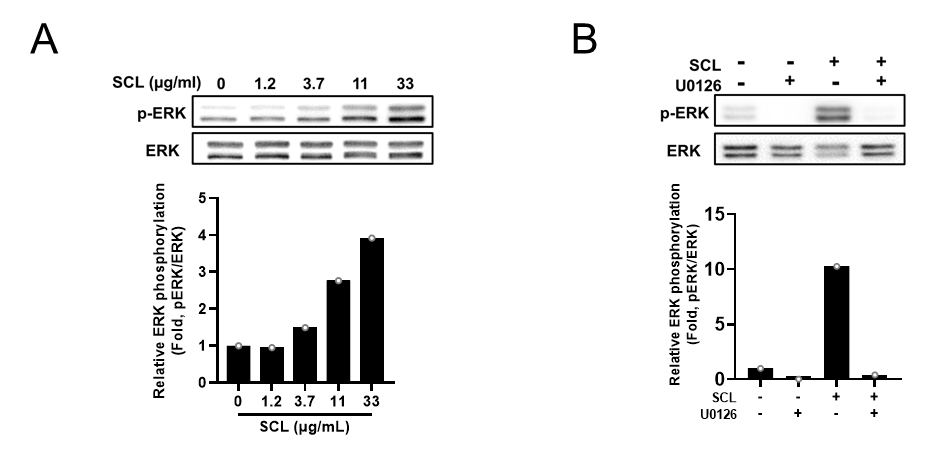

Supplement: S3 Fig — (A) 3T3-L1 preadipocytes were treated with various concentrations of SCL for 15 min. (B) 3T3-L1 preadipocytes were treated with 33-μg/mL SCL for 15 min in the absence or presence of ERK inhibitor U0126 (10 μM). Total ERK and phosphorylated (p)-ERK protein expression levels, as determined by western blotting. (TIF) [file pone.0300520.s003.tif]
