# Supplementary material for: Stellera chamaejasme L. extract inhibits adipocyte differentiation through activation of the extracellular signal-regulated kinase pathway
Source: PLoS One. 2024 Mar 21;19(3):e0300520. doi: 10.1371/journal.pone.0300520 (PMC10956757; doi:10.1371/journal.pone.0300520)
Supplement: S4 Fig — (PDF) [file pone.0300520.s004.pdf]

Original uncropped scan images of Fig. 2B

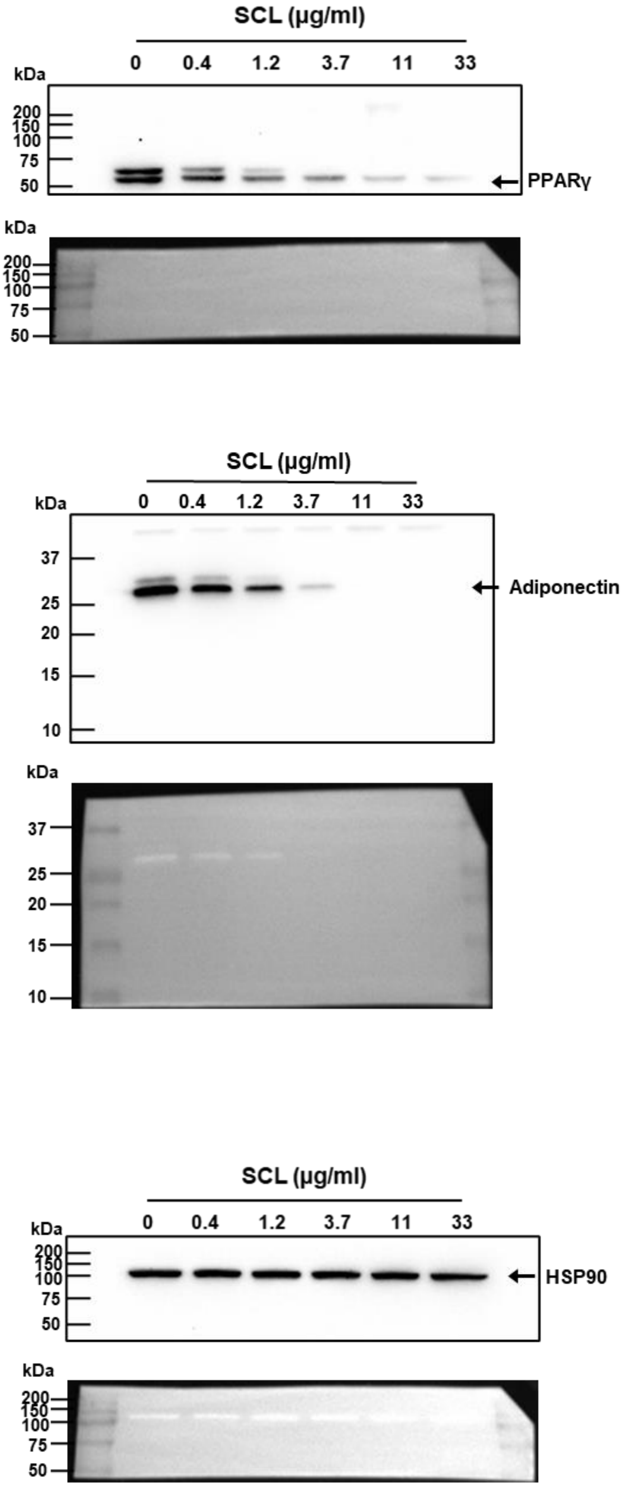

Original uncropped scan images of Fig. 3E

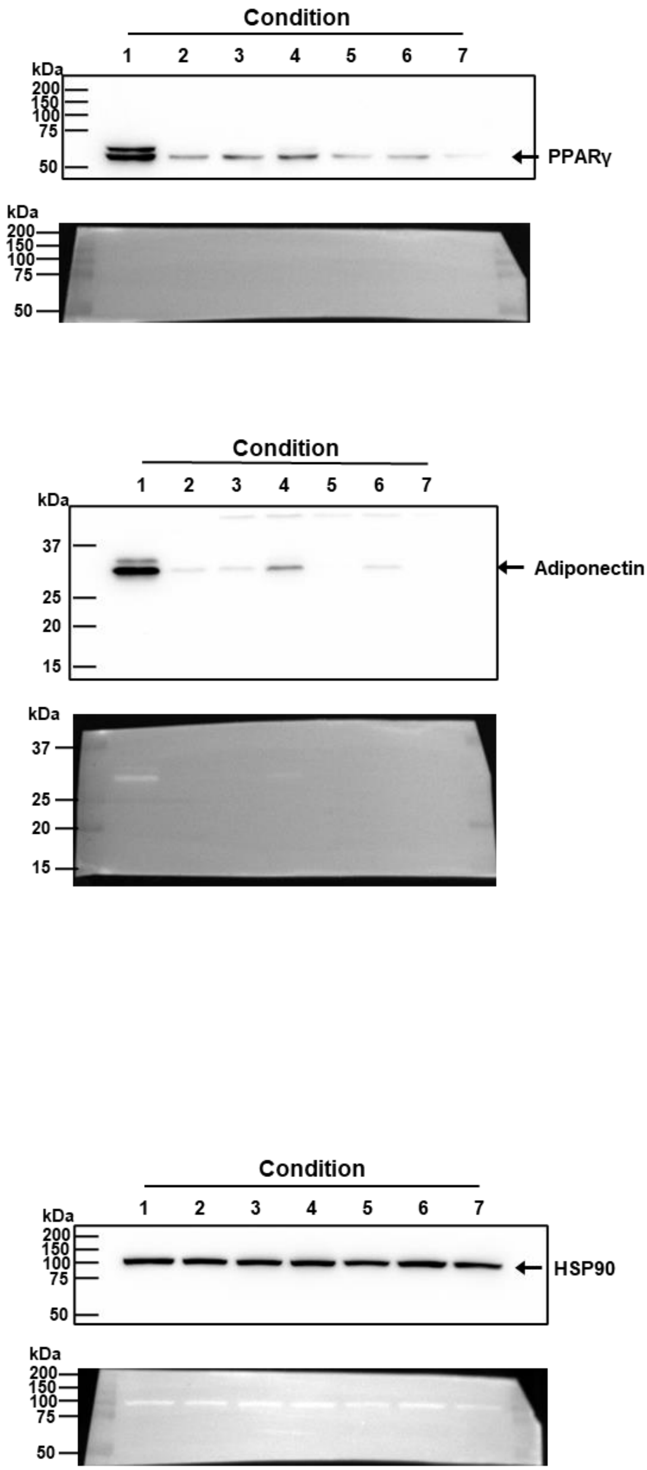

S4 Fig. Original western blot image data. Original uncropped scan images (1/3).

Original uncropped scan images of Fig. 4E

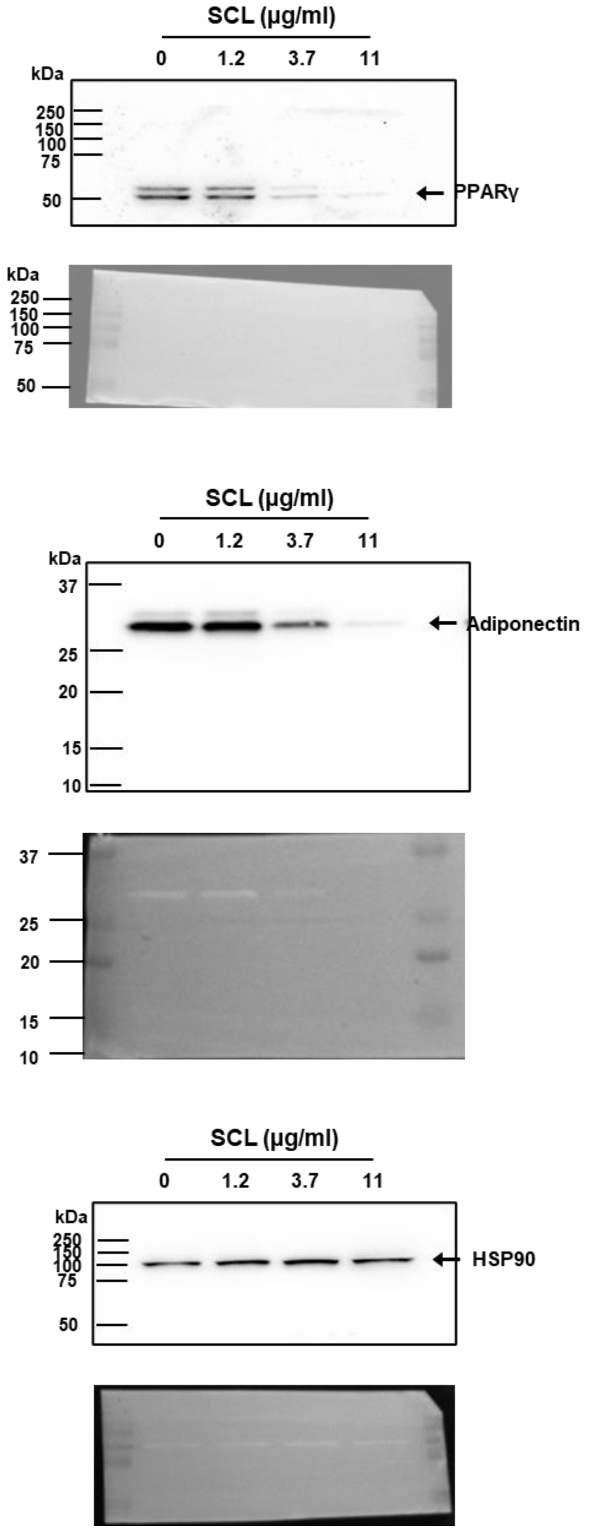

Original uncropped scan images of Fig. 6A

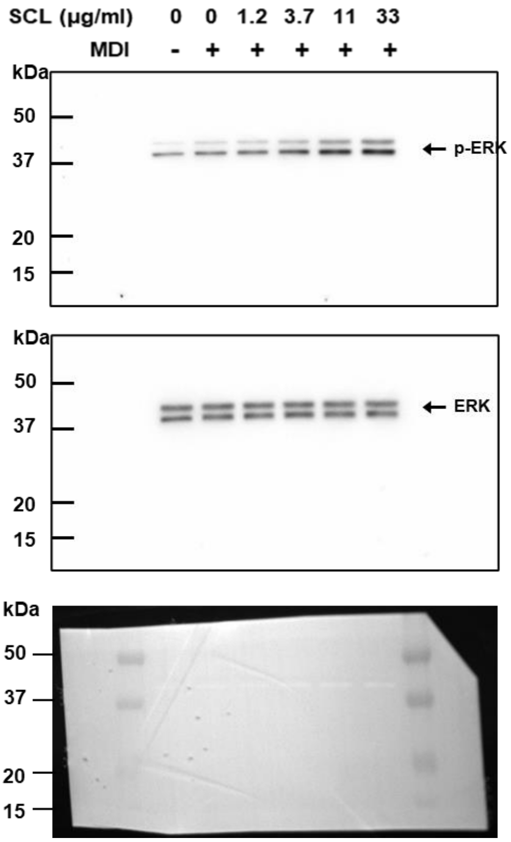

Original uncropped scan images of Fig. 6B

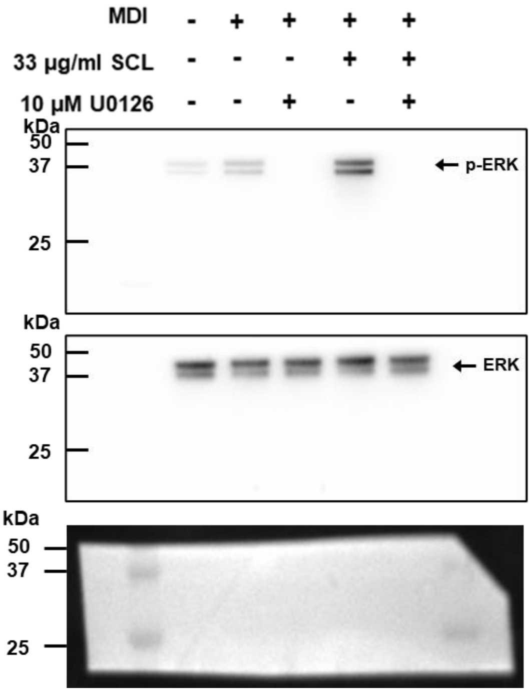

S4 Fig. Original western blot image data. Original uncropped scan images (2/3).

Original uncropped scan images of S3 Fig. A

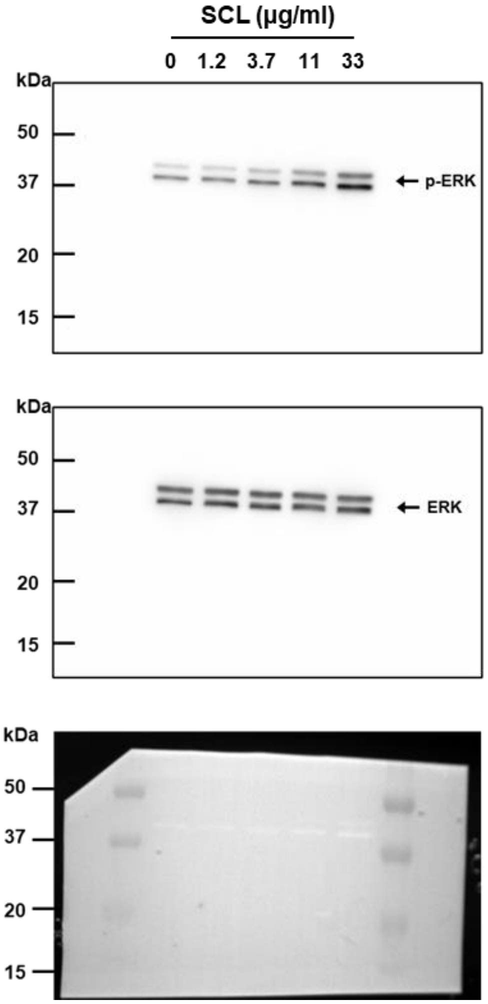

Original uncropped scan images of S3 Fig. B

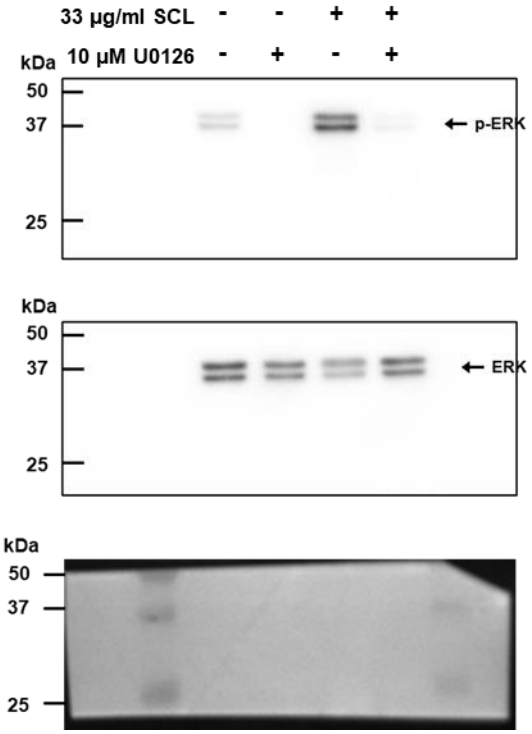

S4 Fig. Original western blot image data. Original uncropped scan images (3/3).
